# Maternal history of miscarriages and measures of fertility in relation to childhood asthma

**DOI:** 10.1136/thoraxjnl-2018-211886

**Published:** 2018-12-04

**Authors:** Maria Christine Magnus, Øystein Karlstad, Christine Louise Parr, Christian M Page, Per Nafstad, Per Magnus, Stephanie J London, Allen J Wilcox, Wenche Nystad, Siri Eldevik Håberg

**Affiliations:** 1 Centre for Fertility and Health (CeFH), Norwegian Institute of Public Health, Oslo, Norway; 2 MRC Integrative Epidemiology Unit, University of Bristol, Bristol, UK; 3 Department of Population Health Sciences, Bristol Medical School, Bristol, UK; 4 Division for Mental and Physical Health, Norwegian Institute of Public Health, Oslo, Norway; 5 Department of Nursing and Health Promotion, OsloMet—Oslo Metropolitan University, Oslo, Norway; 6 Oslo Centre for Biostatistics and Epidemiology, Oslo University Hospital, Oslo, Norway; 7 Department for Community Medicine, Medical Faculty, University of Oslo, Oslo, Norway; 8 Epidemiology Branch, National Institute of Environmental Health Sciences, National Institutes of Health, Research Triangle Park, North Carolina, USA

**Keywords:** asthma epidemiology

## Abstract

**Background:**

It remains unclear what underlies the greater risk of asthma reported among children conceived by assisted reproductive technologies (ART).

**Objective:**

Our aim was to clarify the role of parental subfertility and unmeasured confounding on the association between ART and childhood asthma, and to examine the possibility for common mechanisms underlying parental subfertility and miscarriages influencing asthma pathogenesis.

**Methods:**

We used data from national Norwegian health registries (n=474 402) and the Norwegian Mother and Child Cohort Study (MoBa) (n=75 797). We used log-linear regression to estimate overall associations, and fixed-effects logistic regression to estimate associations within siblings.

**Results:**

ART offspring had greater asthma risk, the adjusted relative risk (aRR) was 1.20 (95% CI 1.09 to 1.32) in the registry-based cohort, and 1.42 (95% CI 1.14 to 1.76) in MoBa. The sibling analysis yielded similar associations, although the CI included the null value. The elevated asthma risk among ART offspring was attenuated when they were compared with spontaneously conceived offspring with time to conception >12 months, aRR 1.22 (95% CI 0.95 to 1.57). Asthma risk also increased with maternal history of early miscarriages (≤12 weeks), with an aRR of 1.07 (95% CI 1.03 to 1.11) for one, aRR 1.18 (95% CI 1.10 to 1.26) for two and aRR 1.24 (95% CI 1.12 to 1.37) for three or more.

**Conclusion:**

Our findings indicate that both parental subfertility and characteristics related to the ART procedure itself might increase offspring asthma risk, although this needs to be confirmed in future studies, and further suggest that common mechanisms underlying parental subfertility and recurrent miscarriages might influence offspring asthma pathogenesis.

**Key messages**

**What is the key question?**

- What is the role for parental subfertility and unmeasured confounding on the association between assisted reproductive technologies (ART) and childhood asthma, and are there common mechanisms underlying parental subfertility and miscarriages influencing asthma pathogenesis?

**What is the bottom line?**

- Our findings indicate that both parental subfertility and characteristics related to the ART procedure itself might increase offspring asthma risk, although this needs to be confirmed in future studies, and further suggest that common mechanisms underlying parental subfertility and recurrent miscarriages might influence offspring asthma pathogenesis.

**Why read on?**

- This population-based study provides novel insight into the underlying mechanisms behind the greater risk of asthma previously reported among children conceived by ART.

## Introduction

An estimated one in four couples globally require assistance to conceive according to WHO.^1^ Assisted reproductive technologies (ART) are associated with greater risk of adverse pregnancy outcomes and may also have long-term consequences for both the mother and child.^2 3^ With regard to whether the risk of asthma is increased in ART offspring, there is inconsistent evidence, with studies reporting both null,^4–6^ and positive findings.^2 7–11^

Only one previous population-based study attempted to distinguish the roles of underlying subfertility (measured by time to conception) and ART in relation to asthma risk.^7^ It therefore remains unclear whether an increased risk of asthma in ART offspring could be explained by characteristics of women who suffer from subfertility, or a direct effect of ART procedures. Furthermore, no previous study compared the risk of asthma among ART offspring with their spontaneously conceived sibling, to examine the role of unmeasured confounding by maternal background characteristics that remain stable between deliveries.^12 13^

There is evidence of common determinants of subfertility and risk of recurrent miscarriages, including immunological mechanisms.^14^ A few previous studies of maternal history of miscarriages and asthma risk suggest a positive association.^15 16^ However, these studies did not distinguish between early versus late miscarriages. Early miscarriages might be more likely to be explained by maternal-fetal incompatibility or chromosomal aberrations.^14^

Our aim was to clarify the role of parental subfertility and unmeasured confounding on the association between ART and childhood asthma, and to examine the possibility for common mechanisms underlying parental subfertility and miscarriages influencing asthma pathogenesis.

## Materials and methods

### Norwegian National Registry Cohort

**Figure 1:**
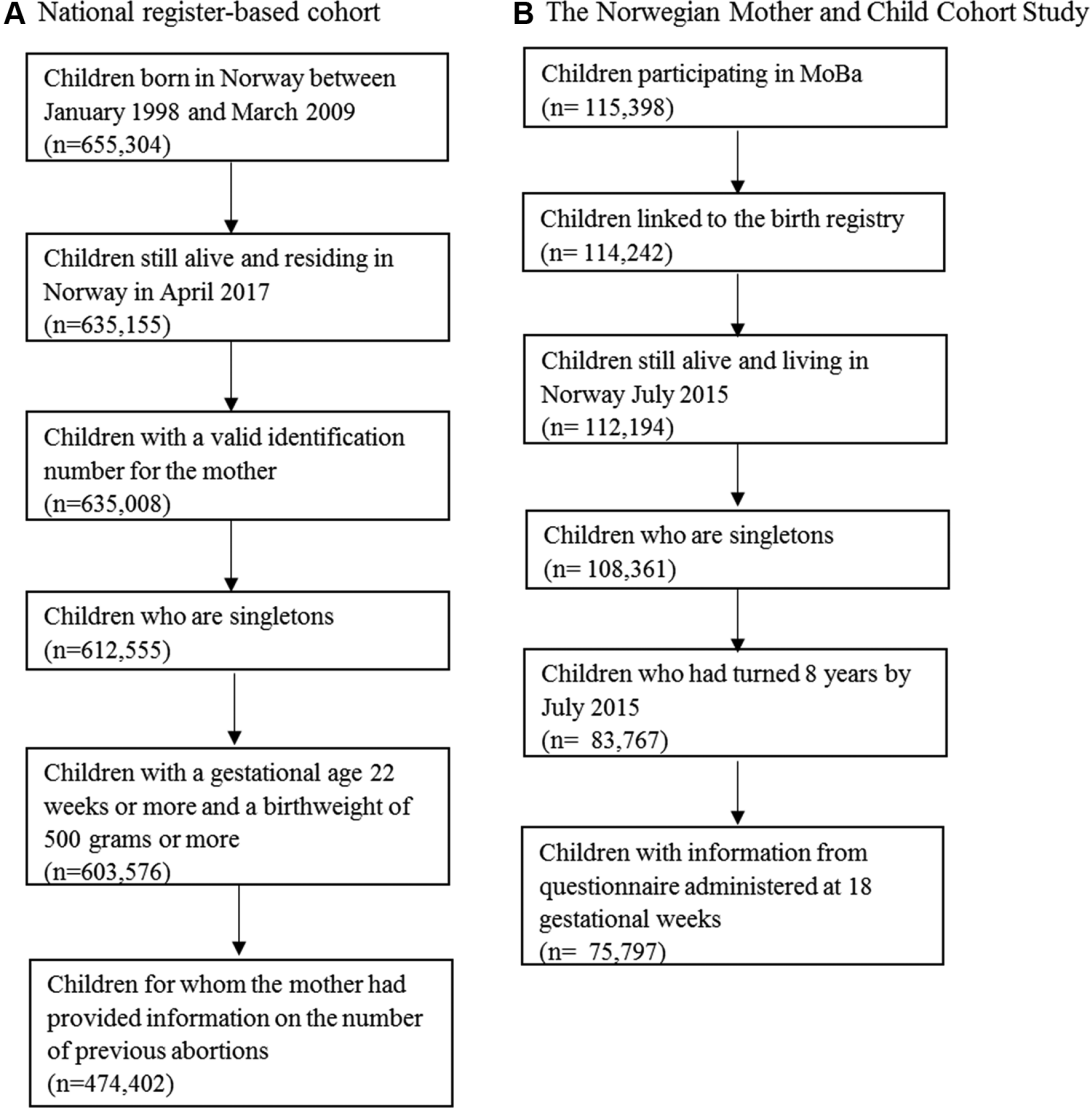
Illustration of sample selection. MoBa, Norwegian Mother and Child Cohort Study.

We linked information from the Medical Birth Registry of Norway (MBRN),^17^ and the Norwegian Prescription Database (NorPD), for all children born in Norway between January 1998 and March 2009. We included children who had reached 8 years by the time of the registry linkage in April 2017. We excluded children who died or emigrated before age 8, those with missing maternal identification number, children from multiple births, children with a birth weight <500 g, children with a gestational age <22 weeks and children with missing exposure information, leaving a total of 474 402 children in the analysis (figure 1A).

### The Norwegian Mother and Child Cohort Study

The Norwegian Mother and Child Cohort Study (MoBa) recruited pregnant women across Norway between 1999 and 2008 at the time of the invitation to the routine ultrasound screening, at approximately gestational week 18.^18 19^ Of the eligible women, 41% participated, and all participants gave a written informed consent. The cohort includes >95 000 women and 114 500 offspring. Notably, all children in MoBa were also included in the registry analysis. We used data available in November 2015 (V.9 of the quality assured data files). Information obtained through questionnaires in MoBa was linked to the MBRN and NorPD. A total of 75 797 singletons in MoBa had reached 8 years by the time of data linkage in July 2015, had information from questionnaires administered at 18 gestational weeks and were included in the current study (figure 1B).

### Assisted reproductive technologies and measures of parental subfertility

The MBRN provided information on whether the offspring was conceived by ART or not (yes vs no). For the register-based cohort, we also examined different subgroups of ART, including in vitro fertilisation (IVF), intracytoplasmic sperm injection (ICSI) and ‘other/unknown’ methods. In MoBa, additional self-reported measures of parental subfertility included previous treatment for unwanted childlessness (yes vs no) and time to conception (<6 months, 6–12 months and >12 months).

### Maternal history of miscarriages

The MBRN included self-reported information on the number of previous miscarriages (0, 1, 2 and 3 or more) the first 12 gestational weeks (early), and the number of miscarriages between 12 and 23 gestational weeks (late). In MoBa, the number of previous miscarriages was self-reported through a questionnaire. The mother reported whether each previous pregnancy had resulted in a live birth, induced abortion or fetal death and the gestational week a fetal death had occurred. A fetal death that occurred during the first 22 gestational weeks was defined as a miscarriage (0, 1, 2 and 3 or more).

### Childhood asthma

We defined childhood asthma using dispensed asthma medications in the NorPD, which is coded according to the Anatomical Therapeutic Chemical Classification System. Asthma medications included inhaled short-acting and long-acting beta(2)-agonists (R03AC), inhaled corticosteroids (R03BA), fixed-dose combinations of inhaled beta(2)-agonists and corticosteroids (R03AK) and leukotriene antagonists (R03DC). We classified children as having asthma at age 7 if at least one prescription for asthma medications was dispensed in the 12 months before the seventh birthday, in addition to a second dispensed prescription within 12 months after the first. In a secondary analysis in MoBa, we defined asthma at age 7 based on maternal report of doctor-diagnosed asthma, in combination with either symptoms or use of asthma medications in the past 12 months.

### Covariates

The MBRN provided information on maternal age at delivery (<25, 26–29, 30–34 and 25 and older), parity (0, 1, 2, 3 or more), pre-eclampsia (yes vs no), in addition to child sex, low birth weight (<2500 g), preterm birth (gestational age <37 completed weeks) and delivery by caesarean section (yes vs no). We used additional information on maternal current asthma (yes vs no), smoking during pregnancy (no, only in the beginning of pregnancy and still smoking at the end of pregnancy) and folate supplement intake (no, started before pregnancy and started during pregnancy) from MBRN for the register-based cohort. For MoBa participants, self-reported information obtained through questionnaires was available for maternal current asthma (yes vs no), smoking (no, quit by 18 gestational weeks and still smoking after 18 gestational weeks), folate intake (no, started before pregnancy and started during pregnancy) and prepregnancy body mass index (<18.5, 18.5–24.9, 25–29.9 and 30 or higher kg/m^2^).

### Statistical analysis

First, we examined the associations of ART, whether the parents had previously been treated for unwanted childlessness, and time to conception, in relation to asthma at age 7. Second, we examined the association between maternal history of miscarriages and risk of childhood asthma. We estimated the associations using log-binomial regression, reporting relative risks (RR) and 95% CIs. We accounted for the dependency between siblings by using robust cluster variance estimation. In the register-based analyses, we adjusted for maternal age, parity and maternal current asthma. We explored additional adjustment for maternal smoking among the 83% of children for whom this information was available. In MoBa, we adjusted for maternal age, parity, education, smoking during pregnancy, prepregnancy body mass index and asthma. We further considered whether the associations of interest were mediated by low birth weight, preterm birth, pre-eclampsia or delivery by caesarean section. These pregnancy complications were conceptualised as potential mediators under the assumption that parental fertility problems and maternal history of miscarriages might increase the risk of adverse pregnancy outcomes in subsequent pregnancies, and that these adverse pregnancy outcomes confer an increased risk of asthma in the offspring (see online supplementary figure S1). We conducted a logistic decomposition^20^ of the direct and indirect effects of the associations of maternal fertility problems and history of miscarriages with offspring asthma according to these adverse pregnancy outcomes. In the register-based cohort, we also compared sibling pairs discordant for asthma, using fixed-effects/conditional logistic regression, reporting ORs and 95% CIs.

10.1136/thoraxjnl-2018-211886.supp1 Supplementary file 1

To examine the role of subfertility as opposed to characteristics of the ART procedure, we compared the risk of asthma among children in MoBa conceived by ART first with spontaneously conceived children born to parents who had tried to conceive for >12 months, and second to children of parents previously treated for unwanted childlessness. We conducted a number of sensitivity analyses. We chose not to adjust for folate supplement intake in the main model, since we did not have strong evidence to support that it might influence the exposures of interest. However, we explored additional adjustment for folate supplement intake in a secondary analysis. Furthermore, we stratified the associations by maternal asthma and preterm birth. Less than 8% of children had missing information on one or more covariates, and we therefore present results from a complete-case analysis. All analyses were conducted using Stata V.14 (StataCorp, College Station, Texas, USA).

## Results

### Distribution of background characteristics

Among the 474 402 children in the register-based cohort, 20 189 (4.3%) had asthma at age 7 defined by dispensed medications. Similarly, among the 75 797 children in the analysis of MoBa, 3229 (4.3%) children had received asthma medications. Mothers of children included in the registry-based analysis were older and less likely to be smokers than those excluded due to missing information on the history of miscarriages (see online supplementary table S1). However, the distribution of adverse pregnancy outcomes and childhood asthma was similar. Children in the register-based cohort had mothers who were younger, more likely to smoke and more likely to have asthma, as compared with the children in MoBa (table 1). The children in the register-based cohort were also more likely to be preterm or low birth weight (table 1). Background characteristics among children in MoBa according to conception by ART, and whether the parents had tried to conceive for more than 12 months, is displayed in online supplementary table S2.

**Table 1:** Distribution of background characteristics

Background characteristics	Registry cohort n=474 402 N (%)	MoBa n=75 797 N (%)
Maternal age at delivery, years		
<25	79 803 (16.8)	8791 (11.6)
25–29	155 866 (32.9)	25 367 (33.5)
30–34	159 001 (33.5)	29 024 (38.3)
35 and older	79 732 (16.8)	12 615 (16.6)
Maternal parity		
0	193 503 (40.8)	33 059 (43.6)
1	170 737 (36.0)	27 291 (36.0)
2	78 163 (16.5)	12 009 (15.8)
3+	31 999 (6.8)	3438 (4.5)
Maternal education		
Less than high school	NA	6698 (8.8)
High school	NA	23 485 (31.0)
Up to 4 years of college	NA	30 223 (39.9)
>4 years of college	NA	15 018 (19.8)
Missing	NA	373 (0.5)
Maternal prepregnancy body mass index		
<18.5	NA	2313 (3.1)
18.5–24.9	NA	47 768 (63.0)
25–29.9	NA	16 391 (21.6)
30 or higher	NA	7122 (9.5)
Missing	NA	2203 (2.9)
Maternal folic acid supplement intake		
No	271 981 (57.3)	16 020 (21.1)
Started before pregnancy	76 333 (16.1)	30 835 (40.7)
Started during pregnancy	124 732 (26.3)	28 942 (38.2)
Missing	1356 (0.3)	0 (0)
Maternal smoking during pregnancy		
No	317 226 (66.9)	56 564 (74.6)
Only at the beginning of pregnancy (register)/quit by 18 weeks (MoBa)	27 929 (5.9)	10 811 (14.3)
Still smoking end of pregnancy (register)/smoked after 18 weeks (MoBa)	48 855 (10.3)	8057 (10.6)
Missing	80 392 (17.0)	365 (0.5)
Maternal current asthma		
No	454 114 (95.7)	73 220 (96.6)
Yes	20 288 (4.3)	2577 (3.4)
Maternal pre-eclampsia		
No	456 100 (96.1)	72 914 (96.2)
Yes	18 302 (3.9)	2883 (3.8)
Child gender		
Male	243 413 (51.3)	38 811 (51.2)
Female	230 989 (48.7)	36 986 (48.8)
Child preterm birth (<37 gestational weeks)		
No	449 218 (94.7)	71 849 (94.8)
Yes	25 184 (5.3)	3646 (4.8)
Missing	0 (0)	302 (0.4)
Child low birth weight (<2500 g)		
No	458 572 (96.7)	73 611 (97.1)
Yes	15 830 (3.3)	2146 (2.8)
Missing	0 (0)	40 (0.1)
Child delivered by caesarean section		
No	404 328 (85.2)	65 288 (86.1)
Yes	70 074 (14.8)	10 509 (13.9)

MoBa, Norwegian Mother and Child Cohort Study.

### Conception by assisted reproductive technologies and measures of parental subfertility in relation to childhood asthma

Children conceived by ART had a higher risk of childhood asthma both in the register-based cohort, adjusted RR 1.20 (95% CI 1.09 to 1.32), and in MoBa, adjusted RR 1.42 (95% CI 1.14 to 1.76) (table 2). The positive associations with asthma were similar across the different subgroups of ART, including IVF, ICSI and ‘other/unspecified’ methods (table 2). The analysis of siblings discordant for childhood asthma in the register-based cohort yielded an association of a similar magnitude with ART, adjusted OR 1.20 (95% CI 0.85 to 1.71), although the CI was widened due to the smaller sample size (table 3). In MoBa, we further observed increased risk of asthma in children of parents previously treated for childlessness, adjusted RR 1.26 (95% CI 1.12 to 1.42), and children of parents who had tried to conceive for >12 months, adjusted RR 1.19 (95% CI 1.06 to 1.34) (table 4). When we compared the risk of asthma among children conceived by ART with spontaneously conceived children of parents who had tried to conceive for >12 months, the adjusted RR was 1.22 (95% CI 0.95 to 1.57). Similarly, comparing ART children with spontaneously conceived children of parents who had previously been treated for unwanted childlessness, the adjusted RR for asthma was 1.23 (95% CI 0.95 to 1.58) (table 5). There was a modest indirect effect of adverse pregnancy outcomes on the association between parental fertility problems/use of ART with offspring asthma risk (see online supplementary table S3 and S4), and the observed associations were therefore mostly not explained by these pathways.

**Table 2:** Association between conception by assisted reproductive technologies and childhood asthma at 7 years

Study population	Conceived by assisted reproductive technologies	N	N cases (%)	Unadjusted RR (95% CI)	Adjusted* RR (95% CI)
Register-based cohort	No	466 034	19 760 (4.2)	1	1
	Yes (total)	8368	429 (5.1)	1.21 (1.10 to 1.33)	1.20 (1.09 to 1.32)
	Yes (IVF)	7339	377 (5.1)	1.21 (1.10 to 1.34)	1.20 (1.08 to 1.33)
	Yes (ICSI)	483	25 (5.2)	1.22 (0.83 to 1.79)	1.21 (0.83 to 1.76)
	Yes (other/unspecified)	546	27 (5.0)	1.17 (0.81 to 1.69)	1.15 (0.79 to 1.65)
MoBa	No	74 406	3144 (4.2)	1	1
	Yes	1391	85 (6.1)	1.45 (1.17 to 1.78)	1.42 (1.14 to 1.76)

*The analysis of the register-based cohort was adjusted for maternal age, parity and maternal current asthma. The analysis of MoBa was adjusted for maternal age, parity, education, smoking during pregnancy, prepregnancy BMI and maternal current asthma.

BMI, body mass index; ICSI, intracytoplasmic sperm injection; IVF, in vitro fertilisation; MoBa, Norwegian Mother and Child Cohort Study; RR, relative risk.

**Table 3:** Sibling analysis of conception by assisted reproductive technologies and childhood asthma at 7 years in the registry-based cohort

Conceived by assisted reproductive technologies	N controls	N cases	Unadjusted OR (95% CI)	Adjusted* OR (95% CI)
No	10 171	8793	1	1
Yes	123	130	1.26 (0.89 to 1.79)	1.20 (0.85 to 1.71)

*Adjusted for maternal age, parity and maternal current asthma.

**Table 4:** Association between measures of parental fertility and childhood asthma at 7 years in the MoBa

Exposure	Exposure group	N	N cases (%)	Unadjusted RR (95% CI)	Adjusted* RR (95% CI)
Previously treated for unwanted childlessness	No	68 011	2827 (4.2)	1	1
	Yes	6018	325 (5.4)	1.30 (1.16 to 1.46)	1.26 (1.12 to 1.42)
Time to conception	<6 months	60 485	2525 (4.2)	1	1
	6–12 months	9431	386 (4.1)	0.98 (0.88 to 1.09)	0.98 (0.88 to 1.09)
	>12 months	5881	318 (5.4)	1.30 (1.16 to 1.45)	1.19 (1.06 to 1.34)

*Adjusted for maternal age, parity, education, smoking during pregnancy, prepregnancy BMI and maternal current asthma.

BMI, body mass index; MoBa, Norwegian Mother and Child Cohort Study; RR, relative risk.

**Table 5:** Comparing the risk of asthma at 7 years among children conceived by assisted reproductive technologies with children of parents who tried to become pregnant for 12 months or were previously treated for unwanted childlessness in the MoBa

Exposure	N	N cases (%)	Unadjusted RR (95% CI)	Adjusted* RR (95% CI)
Previously treated for unwanted childlessness	4653	244 (5.2)	1	1
Conceived by assisted reproductive technologies	1391	85 (6.1)	1.17 (0. 92 to 1.48)	1.23 (0.95 to 1.58)
Time to conception >12 months	4964	262 (5.3)	1	1
Conceived by assisted reproductive technologies	1391	85 (6.1)	1.16 (0.91 to 1.47)	1.22 (0.95 to 1.57)

*Adjusted for maternal age, parity, education, smoking during pregnancy, prepregnancy BMI and maternal current asthma.

BMI, body mass index; MoBa, Norwegian Mother and Child Cohort Study; RR, relative risk.

### Maternal history of miscarriages and childhood asthma

There was a dose-response relationship between risk of childhood asthma and maternal history of miscarriages the first 12 gestational weeks in the register-based analyses, with an adjusted RR of 1.07 (95% CI 1.03 to 1.11) for one, an adjusted RR of 1.18 (95% CI 1.10 to 1.26) for two and an adjusted RR of 1.24 (95% CI 1.12 to 1.37) for three or more (table 6). In contrast, there was no strong evidence of an association with miscarriages between 12 and 23 gestational weeks (table 6). In MoBa, maternal history of miscarriages the first 22 gestational weeks was positively associated with asthma, but the CIs included the null value (table 6). Adverse pregnancy outcomes were found to mediate only a minor part of the association between maternal history of miscarriages and childhood asthma risk (see online supplementary table S5).

**Table 6:** Association between maternal history of miscarriages and childhood asthma at 7 years

Study population	Exposure	Exposure group	N	N cases (%)	Unadjusted RR (95% CI)	Adjusted* RR (95% CI)
Register-based cohort	Number of miscarriages within the first 12 gestational weeks	None	374 866	15 628 (4.2)	1	1
		1	73 323	3256 (4.4)	1.07 (1.03 to 1.11)	1.07 (1.03 to 1.11)
		2	18 415	902 (4.9)	1.17 (1.10 to 1.26)	1.18 (1.10 to 1.26)
		3 or more	7798	403 (5.2)	1.24 (1.12 to 1.37)	1.24 (1.12 to 1.37)
	Number of miscarriages between 12 and 23 gestational weeks	None	448 198	18 979 (4.2)	1	1
		1	11 086	546 (4.9)	1.16 (1.07 to 1.27)	1.21 (1.11 to 1.32)
		2	1273	53 (4.2)	0.98 (0.75 to 1.29)	1.04 (0.79 to 1.36)
		3 or more	418	18 (4.3)	1.02 (0.65 to 1.60)	1.04 (0.66 to 1.65)
MoBa	Number of miscarriages within the first 22 gestational weeks	None	60 982	2564 (4.2)	1	1
		1	11 364	499 (4.4)	1.04 (0.95 to 1.15)	1.06 (0.96 to 1.16)
		2	2555	123 (4.8)	1.14 (0.96 to 1.37)	1.18 (0.98 to 1.41)
		3 or more	896	43 (4.8)	1.14 (0.85 to 1.53)	1.14 (0.85 to 1.53)

*The analysis of the register-based cohort was adjusted for maternal age, parity and maternal current asthma. The analysis of MoBa was adjusted for maternal age, parity, education, smoking during pregnancy, prepregnancy BMI and maternal current asthma.

BMI, body mass index; MoBa, Norwegian Mother and Child Cohort Study; RR, relative risk.

### Sensitivity analyses

When using maternal-reported asthma at age 7 as the outcome, most associations were of similar magnitude as observed in the main analysis (see online supplementary table S6). Additional adjustment for folate supplement intake did not change the findings, nor did adjustment for maternal smoking in the register-based cohort among the subgroup of children with this information available (results not presented). Stratified analyses of the associations by maternal asthma (see online supplementary tables S7-S9) and preterm birth (see online supplementary tables S10-S12) showed no strong evidence of an interaction. Additional multivariable adjustment of the association between maternal history of miscarriages and asthma risk for use of ART did not change the observed associations (see online supplementary table S13).

## Discussion

We observed a higher risk of childhood asthma among children conceived by ART. This increased risk of asthma was not completely explained by a role of parental underlying subfertility, as indicated by our comparison with spontaneously conceived children born to parents who had tried to conceive for >12 months. However, the independent roles of ART procedures from parental underlying subfertility on asthma risk needs to be confirmed in future studies. The higher risk of asthma among ART offspring is unlikely to be explained by unmeasured maternal background characteristics that remained stable between deliveries, as a similar increased risk was observed when we compared children conceived by ART with their spontaneously conceived siblings. We also found a positive dose-response relationship between maternal history of early miscarriages and childhood asthma risk. The incremental increased risk of asthma according to maternal history of early miscarriages, lends some support to the notion that there might be common mechanisms underlying parental subfertility and recurrent miscarriages that play a role in offspring asthma pathogenesis.

### Strengths and limitations

The main strengths of our study include information on several measures of parental fertility. Other benefits included the availability of two datasets with individual strengths, and the possibility of using a sibling comparison to examine the role of unmeasured confounding. With regard to limitations, we relied on maternal report of measures of fertility and history of miscarriages, which might have resulted in misclassification. We cannot exclude the possibility of some bias by selection due to the initial participation rate in MoBa (41%) or by the exclusion of children with missing information on maternal history of miscarriages from the registry linkage (20%). Women who provided information on the number of previous miscarriages to the birth registry were slightly older, had a higher parity, were less likely to smoke and more likely to have a history of asthma. However, as we observed a similar distribution of childhood asthma among those included and excluded from the study, a selection bias is less likely to have influenced the results.^21^ Compared with all women who gave birth in Norway during the recruitment period, participants in MoBa are under-represented by the youngest mothers, women with more than two previous deliveries, smokers and women with a history of stillbirths.^22^ Our findings could also be influenced by ascertainment bias, if mothers who have experience fertility problems might are more likely to take their children to the doctor for respiratory problems. We relied on dispensed asthma medications to define asthma, but we did not have information on whether the medication was actually used. However, maternal report that the child used asthma medications during this age period shows a high concordance with dispensed medications (sensitivity 85% and specificity 98%).^23^ By requiring a second dispensed prescription within 12 months after the first, the probability that medications was not used is reduced. We did not have information on maternal asthma control/symptoms during pregnancy. We therefore cannot exclude the possibility that the severity of maternal asthma symptoms during pregnancy could play a role in the associations of interest.

The point estimates from the main analysis and sibling analysis for the association between ART and asthma were identical in the registry-based cohort, even though the smaller number in the sibling analysis did not provide the power to achieve statistical significance. Sibling analyses are also more vulnerable to confounding by parental background characteristics that change between deliveries.^12^ Examples of parental background characteristics that might have changed between deliveries that we were not able to control for include parental asthma severity, changes in complex lifestyle characteristics (eg, diet) that are difficult to measure accurately and whether the parents moved resulting in different environmental exposures. For these unmeasured characteristics to act as potential confounders, they would have to be associated with both measures of parental fertility and childhood asthma risk.

### Comparison with previous studies

The findings from our study are in line with previous studies reporting a positive association between ART and childhood asthma.^2 7–11^ The number of ART children in previous studies ranged from 59 to 31 918, with relative measures of associations with asthma ranging from an OR of 1.28 to 2.65. One previous study explored a stratified analysis by preterm birth, and found that the positive association between ART and asthma was of a greater magnitude among children born preterm,^10^ which contrasts our findings that the associations were overall of a similar magnitude among children born term and preterm. The UK Millennium Cohort Study is the only previous population-based study that was able to compare the risk of asthma among children conceived by ART with the risk among children of parents who had tried to conceive for >12 months, yielding RRs of 1.98 (95% CI 1.06 to 3.72) and 1.59 (95% CI 0.88 to 2.89) for asthma at age 5 and 7, respectively.^7^

Our study also supports findings from two previous studies that looked at maternal history of miscarriages and risk of child asthma. A Finnish hospital-based study of 40 914 women reported a higher risk of asthma among children of mothers with a history of two or more miscarriages, adjusted OR 1.25 (95% CI 1.04 to 1.51).^15^ Another Finnish register-based study, including 21 038 children with asthma and 21 038 age-matched controls, indicated a positive association between maternal history of at least one miscarriage and asthma risk, adjusted OR 1.17 (95% CI 1.10 to 1.25).^16^ Our study is therefore the first to provide evidence for a positive dose-response relationship between maternal history of miscarriages and asthma risk. Furthermore, we show that the increased risk of asthma in relation to miscarriages is restricted to early miscarriages the first 12 gestational weeks, as we found no evidence to support an association with miscarriages between 12 and 23 gestational weeks.

### Potential explanatory mechanisms

Since we observed associations with different measures of parental fertility, and our results indicate some evidence of an increased risk of asthma among ART children when compared with spontaneously conceived offspring of subfertile parents, we believe that there is evidence for both a role of subfertility and the ART procedures on the risk of childhood asthma. However, it is also possible that the increased risk of asthma among children delivered by ART, when compared with children of parents who tried to become pregnant for >12 months, partly reflects the severity of the underlying subfertility of the parents.

It is plausible that factors related to the ART procedure itself might have a direct effect on risk of asthma, as these procedures may influence the embryo and the fetal environment.^24–26^ Several steps in the ART treatment may alter the natural course of the fetal development, for example, medications taken to induce ovulation and to ensure that the pregnancy stays intact during the early phases, the type of medium used for the culture, freezing and thawing cycles, the possibility of polyspermic fertilisation, the hormonal environment and/or manipulation of the embryo.^24–26^

We propose that common immunological mechanism might plausibly underlie the increased risk of asthma we observed both among children of mothers who suffer from subfertility and miscarriages, since immunological mechanisms contributes to both problems conceiving and repeat pregnancy losses.^27–30^ The T helper (Th)1/Th2 balance has a role in ensuring a successful pregnancy, with an overexpression of Th1 immunity being linked to recurrent miscarriages, whereas Th2 immunity contributes to maternal immune tolerance towards the developing fetus.^28^ Since asthma is a disease with elevated Th2 immunity, it seems less likely that the elevated Th1 immunity in women suffering from subfertility explains our observations. In contrast, the greater levels of Th17 cells found in women with recurrent miscarriages might at least partly explain the associations.^28 29 31 32^ Another potential mechanisms is the expression of killer immunoglobulin-like receptor genes, which seems to play a role in reproductive failure, including the risk of pre-eclampsia, intrauterine growth restriction and recurrent spontaneous abortions.^33^

## Conclusion

Our findings provide some evidence to support that both parental subfertility and characteristics related to the ART procedure might increase offspring asthma risk, although this needs to be confirmed in future studies. Our results also suggest that common mechanisms underlying parental subfertility and recurrent miscarriages might influence offspring asthma pathogenesis.
