# Supplementary material for: Maternal history of miscarriages and measures of fertility in relation to childhood asthma
Source: Thorax. 2018 Dec 4;74(2):106–13. doi: 10.1136/thoraxjnl-2018-211886 (PMC6467238; doi:10.1136/thoraxjnl-2018-211886)
Supplement: Supplementary file 1 [file thoraxjnl-2018-211886supp001.pdf]

Supplementary Figure S1. Illustration of the underlying theoretical framework

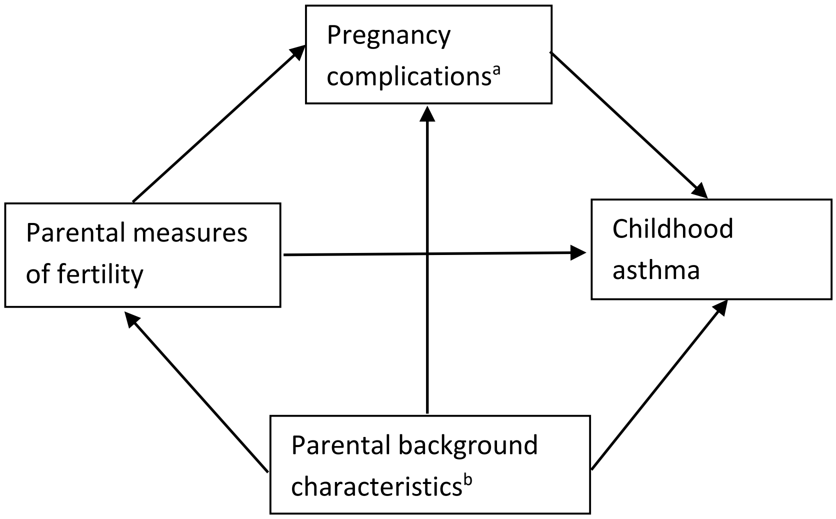

[a] Pregnancy complications evaluated as potential mediators included preterm birth, low birth weight, pre-eclampsia and delivery by caesarean section.

[b] Parental background characteristic evaluated as potential confounders included maternal age, parity, education, smoking during pregnancy, pre-pregnancy body-mass index and asthma.

Supplementary Table S1. Comparing the distribution of background characteristics among children who were included and excluded from the registry-based analysis due to missing exposure information

**Background characteristics**	**Excluded (N=129,174)**	**Included (N=474,402)**
Maternal age at delivery, years N (%)		
<25	24,873 (19.3)	79,803 (16.8)
25-29	45,427 (35.2)	155,866 (32.9)
30-34	40,413 (31.3)	159,001 (33.5)
35 and older	18,461 (14.3)	79,732 (16.8)
Maternal parity, N (%)		
0	53,538 (41.5)	193,503 (40.8)
1	45,311 (35.1)	170,737 (36.0)
2	21,684 (16.8)	78,163 (16.5)
3+	8,641 (6.7)	31,999 (6.8)
Maternal smoking during pregnancy, N (%)		
No	56,162 (78.5)	317,226 (80.5)
Only at the beginning of pregnancy	6,098 (8.5)	27,929 (7.1)
Still smoking at the end of pregnancy	9,294 (13.0)	48,855 (12.4)
Missing	57,620	80,392
Maternal current asthma, N (%)		
No	124,586 (96.5)	454,114 (95.7)
Yes	4,588 (3.6)	20,288 (4.3)
Maternal pre-eclampsia, N (%)		
No	124,414 (96.3)	456,100 (96.1)
Yes	4,760 (3.7)	18,302 (3.9)
Child sex, N (%)		
Male	66,203 (51.3)	243,413 (51.3)
Female	62,971 (48.7)	230,989 (48.7)
Child preterm birth (<37 gestational weeks), N (%)		
No	122,563 (94.9)	449,218 (94.7)
Yes	6,611 (5.1)	25,184 (5.3)
Child low birthweight (<2500 grams), N (%)		
No	125,047 (96.8)	458,572 (96.7)
Yes	4,127 (3.2)	15,830 (3.3)
Missing		
Child delivery by caesarean section, N(%)		
No	112,292 (86.9)	404,328 (85.2)
Yes	16,882 (13.1)	70,074 (14.8)
Child asthma at 7 years, N(%)		
No	123,875 (95.9)	454,213 (95.7)
Yes	5,299 (4.1)	20,189 (4.3)

Supplementary Table S2. Distribution of background characteristics according to time to conception and use of assisted reproductive technologies in the Norwegian Mother and Child Cohort Study
(N= 75,797)

**Background characteristic**	**Time to conception more than 12 months**		**Conceived by assisted reproductive technologies**	
	**No (N=** 69,916**)**	**Yes (N=5,881)**	**No (N=74,406)**	**Yes (N=1,391)**
Maternal age at delivery, Mean(SD)	29.9 (4.6)	31.6 (4.4)	30.0 (4.6)	32.9 (3.8)
Maternal parity, N (%)				
0	29,741 (42.5)	3,318 (56.4)	32,118 (43.2)	941 (67.7)
1	25,496 (36.5)	1,795 (30.5)	26,917 (36.2)	374 (26.9)
2	11,412 (16.3)	597 (10.2)	11,946 (16.1)	63 (4.5)
3+	3,267 (4.7)	171 (2.9)	3,425 (4.6)	13 (0.9)
Maternal education, N (%)				
Less than high school	6,171 (8.8)	527 (9.0)	6,607 (8.9)	91 (6.5)
High school	21,532 (30.8)	1,953 (33.2)	23,084 (31.0)	401 (28.8)
Up to four years of college	27,955 (40.0)	2,268 (38.6)	29,641 (39.8)	582 (41.8)
More than four years of college	13,901 (19.9)	1,117 (19.0)	14,707 (19.8)	311 (22.4)
Missing	357 (0.5)	16 (0.3)	367 (0.5)	6 (0.4)
Maternal pre-pregnancy body-mass index, N (%)				
<18.5	2,132 (3.1)	181 (3.1)	2,283 (3.1)	30 (2.2)
18.5-24.9	44,498 (63.6)	3,270 (55.6)	46,896 (63.0)	872 (62.7)
25-29.9	14,955 (21.4)	1,436 (24.4)	16,066 (21.6)	325 (23.4)
30 or higher	6,233 (8.9)	889 (15.1)	6,985 (9.4)	137 (9.9)
Missing	2,098 (3.0)	105 (1.8)	2,176 (2.9)	27 (1.9)
Maternal folic acid supplement intake				
No	15,053 (21.5)	967 (16.4)	15,927 (21.4)	93 (6.7)
Started before pregnancy	27,944 (40.0)	2,891 (49.2)	29,718 (39.9)	1,117 (80.3)
Started during pregnancy	26,919 (38.5)	2,023 (34.4)	28,761 (38.7)	181 (13.0)
Maternal smoking during pregnancy, N (%)				
No	52,213 (74.7)	4,351 (74.0)	55,358 (74.4)	1,206 (86.7)
Quit by 18 weeks	9,973 (14.3)	838 (14.3)	10,725 (14.4)	86 (6.2)
Smoked after 18 weeks	7,390 (10.6)	667 (11.3)	7,964 (10.7)	93 (6.7)
Missing	340 (0.5)	25 (0.4)	359 (0.5)	6 (0.4)
Maternal current asthma, N (%)				
No	64,658 (92.5)	5,425 (92.3)	71,859 (96.6)	1,361 (97.8)
Yes	5,258 (7.5)	456 (7.8)	2,547 (3.4)	30 (2.2)
Maternal pre-eclampsia, N (%)				
No	67,333 (96.3)	5,581 (94.9)	71,590 (96.2)	1,324 (95.2)
Yes	2,583 (3.7)	300 (5.1)	2,816 (3.8)	67 (4.8)

Child sex, N (%)				
Male	35,784 (51.2)	3,027 (51.5)	38,089 (51.2)	722 (51.9)
Female	34,132 (48.8)	2,854 (48.5)	36,317 (48.8)	669 (48.1)
Child preterm birth (<37 gestational weeks), N (%)				
No	66,413 (95.0)	5,436 (92.4)	70,606 (94.9)	1,243 (89.4)
Yes	3,254 (4.7)	392 (6.7)	3,528 (4.7)	118 (8.5)
Missing	249 (0.4)	53 (0.9)	272 (0.4)	30 (2.2)
Child low birthweight (<2500 grams), N (%)				
No	67,966 (97.2)	5,645 (96.0)	72,294 (97.2)	1,317 (94.7)
Yes	1,914 (2.7)	232 (3.9)	2,073 (2.8)	73 (5.3)
Missing	36 (0.1)	4 (0.1)	39 (0.1)	1 (0.1)
Child delivery by caesarean section, N(%)				
No	60,529 (86.6)	4,759 (80.9)	64,185 (86.3)	1,103 (79.3)
Yes	9,387 (13.4)	1,122 (19.1)	10,221 (13.7)	288 (20.7)

Supplementary Table S3. Examining the direct association between assisted reproductive technologies and childhood asthma at 7 years not mediated by adverse pregnancy outcomes (low birth weight, preterm birth, pre-eclampsia and delivery by caesarean section)

Study population	Conceived by assisted reproductive technologies	Total association OR (95% CI) [a]	Direct association OR (95% CI) [a]	Indirect association OR (95% CI) [a]
Register-based cohort	No	1	1	1
	Yes (total)	1.24 (1.12, 1.36)	1.19 (1.08, 1.31)	1.040 (1.034, 1.045)
	Yes (IVF)	1.24 (1.11, 1.38)	1.19 (1.06, 1.33)	1.039 (1.033, 1.045)
	Yes (ICSI)	1.27 (0.80, 2.02)	1.20 (0.75, 1.92)	1.052 (1.029, 1.076)
	Yes (Other/ unspecified)	1.20 (0.81, 1.78)	1.15 (0.77, 1.72)	1.037 (1.019, 1.057)
MoBa	No	1	1	1
	Yes	1.49 (1.19, 1.87)	1.43 (1.14, 1.80)	1.04 (1.02, 1.05)

[a] The analysis of the register-based cohort was adjusted for maternal age, parity, maternal current asthma and child sex. The analysis of MoBa was adjusted for maternal age, parity, education, smoking during pregnancy, pre-pregnancy BMI, maternal current asthma and child sex.

Supplementary Table S4. Examining the direct associations of parental history of treatment for childlessness and time to conception with childhood asthma at 7 years not mediated by adverse pregnancy outcomes (low birth weight, preterm birth, pre-eclampsia and delivery by caesarean section)

Exposure	Exposure group	Total association OR (95% CI) [a]	Direct association OR (95% CI) [a]	Indirect association OR (95% CI) [a]
Previously treated for unwanted childlessness	No	1	1	1
	Yes	1.31 (1.14, 1.51)	1.28 (1.11, 1.48)	1.03 (1.02, 1.03)
Time to conception	<6 months	1	1	1
	6-12 months	0.97 (0.88, 1.08)	0.97 (0.87, 1.08)	1.00 (0.99, 1.01)
	More than 12 months	1.23 (1.08, 1.39)	1.19 (1.05, 1.35)	1.03 (1.02, 1.04)

[a] Adjusted for maternal age, parity, education, smoking during pregnancy, pre-pregnancy BMI, maternal current asthma and child sex.

Supplementary Table S5. Examining the direct association between maternal history of miscarriages and childhood asthma at 7 years not mediated by adverse pregnancy outcomes (low birth weight, preterm birth, pre-eclampsia and delivery by caesarean section)

Study population	Exposure	Exposure group	Total association OR (95% CI) [a]	Direct association OR (95% CI) [a]	Indirect association OR (95% CI) [a]
Register-based cohort	Number of miscarriages within the first 12 gestational weeks	None	1	1	1
		1	1.07 (1.03, 1.11)	1.06 (1.02, 1.11)	1.004 (1.003, 1.006)
		2	1.20 (1.11, 1.28)	1.18 (1.10, 1.27)	1.012 (1.009, 1.016)
		3 or more	1.26 (1.14, 1.39)	1.21 (1.10, 1.34)	1.037 (1.032, 1.043)
	Number of miscarriages between 12 and 23 gestational weeks	None	1	1	1
		1	1.21 (1.11, 1.31)	1.18 (1.09, 1.28)	1.022 (1.016, 1.027)
		2	1.03 (0.79, 1.33)	0.99 (0.76, 1.28)	1.042 (1.025, 1.058)
		3 or more	1.08 (0.60, 1.94)	1.00 (0.55, 1.80)	1.077 (1.051, 1.104)
MoBa	Number of miscarriages within the first 22 gestational weeks	None	1	1	1
		1	1.06 (0.97, 1.16)	1.06 (0.97, 1.16)	1.00 (0.99, 1.01)
		2	1.18 (0.98, 1.42)	1.17 (0.98, 1.41)	1.01 (0.99, 1.01)
		3 or more	1.12 (0.75, 1.68)	1.10 (0.73, 1.65)	1.02 (1.01, 1.03)

[a] The analysis of the register-based cohort was adjusted for maternal age, parity, maternal current asthma and child sex. The analysis of MoBa was adjusted for maternal age, parity, education, smoking during pregnancy, pre-pregnancy BMI, maternal current asthma and child sex.

Supplementary Table S6. Association between different measures of maternal fertility and childhood asthma at 7 years defined based on maternal report in the Norwegian Mother and Child Cohort Study

Exposure	Exposure group	N	N cases (%)	Unadjusted RR 95% CI	Adjusted [a] RR 95% CI	Adjusted [b] RR 95% CI
Number of miscarriages within 22 completed gestational weeks	None	31,550	1,744 (5.5)	1	1	1
	1	6,015	339 (5.6)	1.02 (0.91, 1.14)	1.04 (0.92, 1.17)	1.04 (0.93, 1.17)
	2	1,345	79 (5.9)	1.06 (0.85, 1.33)	1.08 (0.86, 1.35)	1.07 (0.85, 1.34)
	3 or more	463	28 (6.1)	1.09 (0.76, 1.57)	1.07 (0.75, 1.55)	1.04 (0.73, 1.49)
Previously treated for unwanted childlessness	No	35,224	1,931 (5.5)	1	1	1
	Yes	3,293	214 (6.5)	1.19 (1.03, 1.36)	1.22 (1.06, 1.41)	1.22 (1.06, 1.41)
Time to conception	<6 months	31,112	1,679 (5.4)	1	1	1
	6-12 months	5,046	303 (6.0)	1.11 (0.99, 1.25)	1.11 (0.99, 1.25)	1.11 (0.98, 1.25)
	More than 12 months	3,215	208 (6.5)	1.20 (1.04, 1.38)	1.20 (1.03, 1.38)	1.17 (1.01, 1.35)
Conceived by assisted reproductive technologies	No	38,590	2,141 (5.6)	1	1	1
	Yes	783	49 (6.3)	1.13 (0.86, 1.48)	1.23 (0.93, 1.62)	1.20 (0.91, 1.59)

[a] Adjusted for maternal age, parity, education, smoking during pregnancy, pre-pregnancy BMI and maternal current asthma.

[b] Adjusted for maternal age, parity, education, smoking during pregnancy, pre-pregnancy BMI, maternal current asthma, in addition to child sex, low birthweight, preterm birth, pre-eclampsia and delivery by caesarean sections.

Supplementary Table S7. Association between conception by assisted reproductive technologies and childhood asthma at 7 years stratified by maternal current asthma

Study population	Conceived by assisted reproductive technologies	No maternal current asthma				Maternal current asthma				
		N	N cases (%)	Unadjusted RR 95% CI	Adjusted [a] RR 95% CI	N	N cases (%)	Unadjusted RR 95% CI	Adjusted [a] RR 95% CI	p-value from test for interaction
Register-based cohort	No	446,064	17,616 (4.0)	1	1	19,970	2,144 (10.7)	1	1	0.11
	Yes (total)	8,050	397 (4.9)	1.25 (1.13, 1.38)	1.23 (1.12, 1.36)	318	32 (10.1)	0.94 (0.67, 1.30)	0.90 (0.65, 1.26)	
	Yes (IVF)	7,066	353 (5.0)	1.27 (1.14, 1.40)	1.25 (1.12, 1.39)	273	24 (8.8)	0.82 (0.56, 1.20)	0.78 (0.54, 1.16)	0.07
	Yes (ICSI)	462	20 (4.3)	1.10 (0.71, 1.68)	1.08 (0.70, 1.66)	21	5 (23.8)	2.22 (1.06, 4.65)	2.10 (0.99, 4.45)	
	Yes (Other/ unspecified)	522	24 (4.6)	1.16 (0.79, 1.72)	1.15 (0.78, 1.70)	24	3 (12.5)	1.16 (0.40, 3.38)	1.12 (0.38, 3.27)	
MoBa	No	71,859	2,831 (3.9)	1	1	2,547	313 (12.3)	1	1	0.52
	Yes	1,361	80 (5.9)	1.49 (1.20, 1.85)	1.45 (1.16, 1.81)	30	5 (16.7)	1.36 (0.61, 3.04)	1.00 (0.39, 2.54)	

[a] The analysis of the register-based cohort was adjusted for maternal age, parity, and maternal current asthma. The analysis of MoBa was adjusted for maternal age, parity, education, smoking during pregnancy, pre-pregnancy BMI and maternal current asthma.

Supplementary Table S8. Association between measures of parental fertility and childhood asthma at 7 years in the Norwegian Mother and Child Cohort Study stratified by maternal current asthma

Exposure	Exposure group	No maternal current asthma				Maternal current asthma				
		N	N cases (%)	Unadjusted RR 95% CI	Adjusted [a] RR 95% CI	N	N cases (%)	Unadjusted RR 95% CI	Adjusted [a] RR 95% CI	p-value from test for interaction
Previously treated for unwanted childlessness	No	65,683	2,545 (3.9)	1	1	2,328	282 (12.1)	1	1	0.46
	Yes	5,825	299 (5.1)	1.32 (1.18, 1.49)	1.29 (1.14, 1.46)	193	26 (13.5)	1.11 (0.76, 1.63)	1.04 (0.71, 1.53)	
Time to conception	<6 months	58,409	2,272 (3.9)	1	1	2,076	253 (12.2)	1	1	0.28
	6-12 months	9,136	346 (3.8)	0.97 (0.87, 1.09)	0.97 (0.86, 1.08)	295	40 (13.6)	1.11 (0.81, 1.52)	1.06 (0.78, 1.46)	
	More than 12 months	5,675	293 (5.2)	1.33 (1.18, 1.49)	1.23 (1.09, 1.39)	206	25 (12.1)	1.00 (0.68, 1.46)	0.86 (0.57, 1.30)	

[a] Adjusted for maternal age, parity, education, smoking during pregnancy, pre-pregnancy BMI and maternal current asthma.

Supplementary Table S9. Association between maternal history of miscarriages and childhood asthma at 7 years stratified by maternal current asthma

Study population	Exposure	Exposure group	No maternal current asthma				Maternal current asthma				
			N	N cases (%)	Unadjusted RR 95% CI	Adjusted [a] RR 95% CI	N	N cases (%)	Unadjusted RR 95% CI	Adjusted [a] RR 95% CI	p-value from test for interaction
Register-based cohort	Number of miscarriages within the first 12 gestational weeks	None	359,367	13,971 (3.9)	1	1	15,499	1,657 (10.7)	1	1	0.48
		1	69,864	2,895 (4.1)	1.07 (1.02, 1.11)	1.08 (1.04, 1.12)	3,459	361 (10.4)	0.98 (0.87, 1.09)	0.96 (0.86, 1.08)	
		2	17,506	798 (4.6)	1.17 (1.09, 1.26)	1.20 (1.12, 1.29)	909	104 (11.4)	1.07 (0.89, 1.29)	1.04 (0.86, 1.26)	
		3 or more	7,377	349 (4.7)	1.22 (1.10, 1.35)	1.25 (1.12, 1.39)	421	54 (12.8)	1.20 (0.93 (1.55)	1.16 (0.89, 1.50)	
	Number of miscarriages between 12 and 23 gestational weeks	None	429,158	16,941 (4.0)	1	1	19,040	2,038 (10.7)	1	1	0.66
		1	10,595	484 (4.6)	1.16 (1.06, 1.27)	1.22 (1.11, 1.33)	491	62 (12.6)	1.18 (0.92, 1.51)	1.15 (0.90, 1.48)	
		2	1,224	50 (4.1)	1.03 (0.78, 1.36)	1.10 (0.83, 1.46)	49	3 (6.1)	0.57 (0.19, 1.72)	0.54 (0.18, 1.65)	
		3 or more	396	15 (3.8)	0.96 (0.58, 1.58)	1.01 (0.61, 1.67)	22	3 (13.6)	1.27 (0.44, 3.68)	1.19 (0.41, 3.47)	
MoBa	Number of miscarriages within the first 22 gestational weeks	None	58,901	2,314 (3.9)	1	1	2,081	250 (12.0)	1	1	0.50
		1	11,008	456 (4.1)	1.05 (0.96, 1.16)	1.06 (0.96, 1.18)	356	43 (12.1)	1.01 (0.74, 1.36)	1.01 (0.74, 1.37)	
		2	2,454	105 (4.3)	1.09 (0.90, 1.32)	1.14 (0.94, 1.38)	101	18 (17.8)	1.48 (0.94, 2.34)	1.45 (0.92, 2.30)	
		3 or more	857	36 (4.2)	1.07 (0.77, 1.48)	1.09 (0.79, 1.51)	39	7 (18.0)	1.49 (0.77, 2.90)	1.42 (0.69, 2.92)	

[a] The analysis of the register-based cohort was adjusted for maternal age, parity, and maternal current asthma. The analysis of MoBa was adjusted for maternal age, parity, education, smoking during pregnancy, pre-pregnancy BMI and maternal current asthma.

[b] Additional adjustment child sex, low birthweight, preterm birth, pre-eclampsia and delivery by caesarean section.

Supplementary Table S10. Association between conception by assisted reproductive technologies and childhood asthma at 7 years stratified by preterm birth

Study population	Conceived by assisted reproductive technologies	Term				Preterm				
		N	N cases (%)	Unadjusted RR 95% CI	Adjusted [a] RR 95% CI	N	N cases (%)	Unadjusted RR 95% CI	Adjusted [a] RR 95% CI	p-value from test for interaction
Register-based cohort	No	441,516	18,041 (4.1)	1	1	24,518	1,719 (7.0)	1	1	0.68
	Yes (total)	7,702	372 (4.8)	1.18 (1.07, 1.31)	1.17 (1.06, 1.30)	666	57 (8.6)	1.22 (0.95, 1.57)	1.22 (0.94, 1.58)	
	Yes (IVF)	6,753	325 (4.8)	1.18 (1.06, 1.31)	1.17 (1.05, 1.30)	586	52 (8.9)	1.27 (0.97, 1.65)	1.27 (0.97, 1.66)	0.39
	Yes (ICSI)	445	24 (5.4)	1.32 (0.89, 1.95)	1.31 (0.89, 1.92)	38	1 (2.6)	0.37 (0.05, 2.60)	0.39 (0.06, 2.49)	
	Yes (Other/ unspecified)	504	23 (4.6)	1.12 (0.75, 1.67)	1.11 (0.74, 1.65)	42	4 (9.5)	1.36 (0.53, 3.45)	1.27 (0.49, 3.30)	
MoBa	No	70,606	2,904 (4.1)	1	1	3,528	233 (6.6)	1	1	0.34
	Yes	1,243	71 (5.7)	1.39 (1.11, 1.74)	1.35 (1.07, 1.70)	118	13 (11.0)	1.67 (0.98, 2.83)	1.77 (1.03, 3.06)	

[a] The analysis of the register-based cohort was adjusted for maternal age, parity, and maternal current asthma. The analysis of MoBa was adjusted for maternal age, parity, education, smoking during pregnancy, pre-pregnancy BMI and maternal current asthma.

Supplementary Table S11. Association between measures of parental fertility and childhood asthma at 7 years in the Norwegian Mother and Child Cohort Study stratified by preterm birth

Exposure	Exposure group	Term				Preterm				
		N	N cases (%)	Unadjusted RR 95% CI	Adjusted [a] RR 95% CI	N	N cases (%)	Unadjusted RR 95% CI	Adjusted [a] RR 95% CI	p-value from test for interaction
Previously treated for unwanted childlessness	No	64,598	2,614 (4.1)	1	1	3,173	206 (6.5)	1	1	0.88
	Yes	5,575	290 (5.2)	1.29 (1.14, 1.45)	1.26 (1.11, 1.42)	390	34 (8.7)	1.34 (0.94, 1.91)	1.28 (0.87, 1.86)	
Time to conception	<6 months	57,466	2,339 (4.1)	1	1	2,804	182 (6.5)	1	1	0.77
	6-12 months	8,947	355 (4.0)	0.97 (0.87, 1.09)	0.97 (0.87, 1.08)	450	29 (6.4)	0.99 (0.68, 1.45)	1.02 (0.70, 1.50)	
	More than 12 months	5,436	281 (5.2)	1.27 (1.13, 1.43)	1.16 (1.03, 1.32)	392	35 (8.9)	1.38 (0.98, 1.94)	1.31 (0.91, 1.90)	

[a] Adjusted for maternal age, parity, education, smoking during pregnancy, pre-pregnancy BMI and maternal current asthma.

Supplementary Table S12. Association between maternal history of miscarriages and childhood asthma at 7 years stratified by preterm birth

Study population	Exposure	Exposure group	Term				Preterm				
			N	N cases (%)	Unadjusted RR 95% CI	Adjusted [a] RR 95% CI	N	N cases (%)	Unadjusted RR 95% CI	Adjusted [a] RR 95% CI	p-value from test for interaction
Register-based cohort	Number of miscarriages within the first 12 gestational weeks	None	355,532	14,328 (4.0)	1	1	19,334	1,300 (6.7)	1	1	0.27
		1	69,280	2,938 (4.2)	1.05 (1.01, 1.09)	1.05 (1.01, 1.10)	4,043	318 (7.9)	1.17 (1.04, 1.32)	1.15 (1.02, 1.29)	
		2	17,272	805 (4.7)	1.16 (1.08, 1.24)	1.16 (1.08, 1.25)	1,143	97 (8.5)	1.26 (1.04, 1.54)	1.24 (1.02, 1.51)	
		3 or more	7,134	342 (4.8)	1.19 (1.07, 1.32)	1.19 (1.07, 1.33)	664	61 (9.2)	1.37 (1.06, 1.75)	1.32 (1.02, 1.70)	
	Number of miscarriages between 12 and 23 gestational weeks	None	424,857	17,352 (4.1)	1	1	23,341	1,627 (7.0)	1	1	0.44
		1	10,227	471 (4.6)	1.13 (1.03, 1.24)	1.18 (1.07, 1.29)	859	75 (8.7)	1.25 (1.00, 1.56)	1.21 (0.96, 1.52)	
		2	1,152	48 (4.2)	1.02 (0.77, 1.35)	1.09 (0.83, 1.45)	121	5 (4.1)	0.60 (0.25, 1.40)	0.55 (0.24, 1.30)	
		3 or more	364	15 (4.1)	1.01 (0.61, 1.66)	1.06 (0.64, 1.75)	54	3 (5.6)	0.80 (0.26, 2.40)	0.71 (0.25, 2.06)	
MoBa	Number of miscarriages within the first 22 gestational weeks	None	57,867	2,379 (4.1)	1	1	2,869	179 (6.2)	1	1	0.13
		1	10,748	449 (4.2)	1.02 (0.92, 1.12)	1.03 (0.93, 1.14)	575	49 (8.5)	1.37 (1.00, 1.86)	1.47 (1.06, 2.04)	
		2	2,401	111 (4.6)	1.12 (0.93, 1.36)	1.16 (0.96, 1.40)	143	11 (7.7)	1.23 (0.69, 2.21)	1.27 (0.69, 2.34)	
		3 or more	833	36 (4.3)	1.05 (0.76, 1.45)	1.06 (0.77, 1.47)	59	7 (11.9)	1.90 (0.95, 3.81)	1.90 (1.08, 3.36)	

[a] The analysis of the register-based cohort was adjusted for maternal age, parity, and maternal current asthma. The analysis of MoBa was adjusted for maternal age, parity, education, smoking during pregnancy, pre-pregnancy BMI and maternal current asthma.

[b] Additional adjustment child sex, low birthweight, preterm birth, pre-eclampsia and delivery by caesarean section.

Supplementary Table S13. Exploring the additional adjustment for use of assisted reproductive technologies for the association between maternal history of miscarriages and childhood asthma at 7 years

Study population	Exposure	Exposure group	N	N cases (%)	Unadjusted RR 95% CI	Adjusted [a] RR 95% CI	Adjusted [b] RR 95% CI
Register-based cohort	Number of miscarriages within the first 12 gestational weeks	None	374,866	15,628 (4.2)	1	1	1
		1	73,323	3,256 (4.4)	1.07 (1.03, 1.11)	1.07 (1.03, 1.11)	1.07 (1.03, 1.11)
		2	18,415	902 (4.9)	1.17 (1.10, 1.26)	1.18 (1.10, 1.26)	1.18 (1.10, 1.26)
		3 or more	7,798	403 (5.2)	1.24 (1.12, 1.37)	1.24 (1.12, 1.37)	1.24 (1.12, 1.36)
	Number of miscarriages between 12 and 23 gestational weeks	None	448,198	18,979 (4.2)	1	1	1
		1	11,086	546 (4.9)	1.16 (1.07, 1.27)	1.21 (1.11, 1.32)	1.20 (1.10, 1.31)
		2	1,273	53 (4.2)	0.98 (0.75, 1.29)	1.04 (0.79, 1.36)	1.03 (0.79, 1.35)
		3 or more	418	18 (4.3)	1.02 (0.65, 1.60)	1.04 (0.66, 1.65)	1.04 (0.66, 1.64)
MoBa	Number of miscarriages within the first 22 gestational weeks	None	60,982	2,564 (4.2)	1	1	1
		1	11,364	499 (4.4)	1.04 (0.95, 1.15)	1.06 (0.96, 1.16)	1.06 (0.96, 1.16)
		2	2,555	123 (4.8)	1.14 (0.96, 1.37)	1.18 (0.98, 1.41)	1.18 (0.98, 1.41)
		3 or more	896	43 (4.8)	1.14 (0.85, 1.53)	1.14 (0.85, 1.53)	1.13 (0.84, 1.51)

[a] The analysis of the register-based cohort was adjusted for maternal age, parity, and maternal current asthma. The analysis of MoBa was adjusted for maternal age, parity, education, smoking during pregnancy, pre-pregnancy BMI and maternal current asthma.

[b] Additional adjustment for use of assisted reproductive technologies.
